# Increased Plasma Levels of Heat Shock Protein 70 Associated with Subsequent Clinical Conversion to Mild Cognitive Impairment in Cognitively Healthy Elderly

**DOI:** 10.1371/journal.pone.0119180

**Published:** 2015-03-13

**Authors:** Sang Joon Son, Kang Soo Lee, Ji Hyung Chung, Ki Jung Chang, Hyun Woong Roh, Soo Hyun Kim, Taewon Jin, Joung Hwan Back, Hyun Jung Kim, Yunhwan Lee, Seong Hye Choi, Jai Sung Noh, Ki Young Lim, Young Ki Chung, Chang Hyung Hong, Byoung Hoon Oh

**Affiliations:** 1 Department of Psychiatry, Ajou University School of Medicine, 164 Worldcup-ro, Yeongtong-gu, Suwon-si, Gyeonggi-do 443–380, Republic of Korea; 2 Department of Psychiatry, CHA Gangnam Medical Center, CHA University, Nonhyon-ro, Gangnam-gu, Seoul 135–081, Republic of Korea; 3 Department of Applied Bioscience, College of Life Science, CHA University, Yatap-dong, Bundang-gu, Seongnam-si, Gyeonggi-do 463–400, Republic of Korea; 4 Graduate Program in Science for Aging, Yonsei University, 50 Yonsei-ro, Seodaemun-gu, Seoul 120–752, Republic of Korea; 5 Health Insurance Policy Research Institute, National Health Insurance Service, 311 Dongmak-ro, Mapo-gu, Seoul 121–749, Republic of Korea; 6 Department of Psychiatry, National Medical Center, 245 Eulji-ro, Jung-gu, Seoul 100–799, Republic of Korea; 7 Department of Preventive Medicine and Public Health, Ajou University School of Medicine, 164 Worldcup-ro, Yeongtong-gu, Suwon-si, Gyeonggi-do 443–380, Republic of Korea; 8 Department of Neurology, Inha University College of Medicine, 27 Inhang-ro, Jung-gu, Incheon 400–711, Republic of Korea; 9 Department of Psychiatry and Institute of Behavioral Science in Medicine, Yonsei University College of Medicine, 50 Yonsei-ro, Seodaemun-gu, Seoul 120–752, Republic of Korea; Taipei Veterans General Hospital, TAIWAN

## Abstract

**Background and Aims:**

Heat shock proteins (HSPs) have been regarded as cytoprotectants that protect brain cells during the progression of neurodegenerative diseases and from damage resulting from cerebral ischemia. In this study, we assessed the association between plasma HSP 70/27 levels and cognitive decline.

**Methods:**

Among participants in the community-based cohort study of dementia called the Gwangju Dementia and Mild Cognitive Impairment Study, subjects without cognitive impairment at baseline, who then either remained without impairment (non-conversion group), or suffered mild cognitive impairment (MCI) (conversion group) (non-conversion group, N = 36; conversion group, N = 30) were analyzed.

**Results:**

After a five to six year follow-up period, comparison of the plasma HSP 70 and HSP 27 levels of the two groups revealed that only the plasma HSP 70 level was associated with a conversion to MCI after adjustments for age, gender, years of education, follow-up duration, APOE e4, hypertension, and diabetes (repeated measure analysis of variance: F = 7.59, *p* = 0.008). Furthermore, an increase in plasma HSP 70 level was associated with cognitive decline in language and executive function (linear mixed model: Korean Boston Naming Test, -0.426 [-0.781, -0.071], *p* = 0.019; Controlled Oral Word Association Test, -0.176 [-0.328, -0.023], *p* = 0.024; Stroop Test, -0.304 [-0.458, -0.150], *p*<0.001).

**Conclusions:**

These findings suggest that the plasma HSP 70 level may be related to cognitive decline in the elderly.

## Introduction

Heat shock proteins (HSPs) are expected to be critically involved in the progression of neurodegeneration and potential therapeutic targets for cognitive decline in the elderly [1,2]. HSPs are molecular chaperones providing a first line of defense against misfolded proteins, a characteristic pathology of neurodegenerative dementia [3]. HSPs also interfere with detrimental processes that occur during neurodegeneration, including oxidative stress and abnormal activation of signaling pathways [4–6]. In that context, early synaptic and axonal abnormalities in neurodegenerative diseases such as Alzheimer’s disease (AD) may therefore be early detected or partially reversed by HSPs [7,8].

Among HSPs, the expression of two HSPs, namely, the 70-kDa HSP (HSP 70) and 27-kDa HSP (HSP 27), in the brain is notable because both are highly induced in glial cells and neurons following various of noxious stimuli in the early neurodegeneration stage [9]. Several studies reported that HSP 70 and HSP 27 levels are increased in subjects with mild cognitive impairment (MCI), a transitional stage between normal aging and dementia. Both of these HSPs are known to have neuroprotective effects in the brain due to their anti-apoptotic and chaperoning activities [2,10].

However, it remains unclear whether circulating concentrations of these HSPs change as a person gets older, and whether there are differences in the HSPs plasma levels according to the presence of cognitive impairment. In addition, to our knowledge, there is no published data on HSPs level-related cognitive phenotypes even though the association between HSPs and cognitive impairment in the elderly has been reported [9]. When considering early diagnostic and therapeutic applications for HSP 70 and HSP 27, it is important to note the specific response of HSPs according to time and cognitive phenotype which reflect the progression and severity of regional brain pathology. Thus, we investigated the longitudinal associations among plasma HSP 70/27 levels, the conversion from no cognitive impairment (NCI) to MCI, and cognitive phenotype in the community-dwelling elderly.

## Materials and Methods

### 1. Subjects

This study was based on data derived from a community-based cohort study called the Gwangju Dementia and Mild Cognitive Impairment Study (GDEMCIS), which was designed to assess the occurrence and risk factors of dementia. We recruited elderly subjects over the age of 60 who resided within a well-defined geographic region in a small city with agricultural districts. Detailed descriptions of GDEMCIS have been provided in prior literature [10,11]. Of the GDEMCIS participants, we retrospectively studied 66 subjects who had 1) normal cognitive function in baseline assessment, 2) normal cognitive function or MCI at a follow-up assessment 3) complete baseline/follow-up cognitive test data, 4) baseline/follow-up blood samples, 5) no newly developed physical illness to be treated from baseline status and 6) no history of taking saclicylate prophylaxis which is a known inducer of HSP 70 in several tissues [12]. All subjects included in this study had clinical data of two time points; at baseline and at the follow-up assessment, and were classified into a non-conversion group (NCI to NCI) or conversion group (NCI to MCI). The NCI group was defined as the one with each cognitive domain (memory, language, visuospatial, and frontal/executive function) test score higher than the cutoff values. The cutoff values for each factor score were 1.0 standard deviation (SD) of the age-adjusted mean for neuropsychological tests assessing cognitive domains. All NCI subjects were classified as Clinical Dementia Rating (CDR) 0. The diagnostic criteria of MCI included the following: 1) subjective cognitive decline that were corroborated by an informant, 2) objective impairments that were 1.0 SD below the age-adjusted mean for at least one of the neuropsychological tests assessing the four cognitive domains, 3) activities of daily living were preserved, and 4) CDR was 0.5. None of our subjects met any of the following exclusion criteria during each visit: 1) a history of significant hearing or visual impairment hindering participation in the interview, 2) neurological disorders (e.g., stroke, Parkinson’s disease or active epilepsy), 3) psychiatric disorders (e.g., schizophrenia, mental retardation, severe depression or, mania), 4) history of use of psychotropic medications, or a history of use of psychoactive substances other than alcohol, and 5) physical illnesses or disorders that could interfere with the clinical study such as severe cardiac disorders, severe respiratory illnesses, uncontrolled diabetes, uncontrolled hypertension, malignancy, and hepatic or renal disorders. All subjects provided their written, informed consent after providing a complete description of the study to the subjects and their relatives. This study was approved by the Institutional Review Board of Severance Mental Hospital.

### 2. Assessments and measurements

The clinical evaluation form was filled out by informants after they had received appropriate instructions. The clinical evaluation form included questions related to: 1) basic demographic characteristics and history of cognitive decline, 2) Korean version of the Mini-Mental State Examination [13], 3) CDR [14], 4) the 15–item geriatric depression scale [15], and 5) a medical history including vascular risk factors such as hypertension and diabetes. Medical diseases were regarded as positive if the patient had either previously been diagnosed with the associated disease or was currently under medical treatment for the disease.

A standardized neuropsychological battery, the Seoul Neuropsychological Screening Battery-Dementia Version (SNSB-D) was used to assess all of the participants [16]. The SNSB-D includes tests of verbal memory, language, visuospatial function, and frontal/executive function domains. These domains were assessed by performing the following tests: the Seoul Verbal Learning Test-delayed recall (SVLT-delayed recall) for verbal memory, the Controlled Oral Word Association Test (COWAT), the Stroop Test-color reading for frontal/executive function, the Korean short version of the Boston Naming Test (K-BNT) for language, and the Rey-Complex Figure Test-copy (RCFT-copy) for visuospatial function.

All blood samples were drawn in the morning from consenting subjects after an eight-hour fast. Blood samples were not obtained from subjects who were in the acute phase of an illness or had a history of fever, alcohol drinking, exercise, or severe stress within the previous week. Plasma samples obtained from all study subjects were aliquoted and stored at-80°C until analysis. Plasma HSP 70 and HSP 27 levels were analyzed using commercially available enzyme-linked immunosorbent assay (ELISA) kits (StressGen, Victoria, Canada). The average intra-assay coefficient of variation was 6% and the average inter-assay coefficient of variation was 9% for HSP 70 ELISA assay. The average intra-assay coefficient of variation was 7% and the average inter-assay coefficient of variation was 8% for HSP 27 ELISA assay.

### 3. Statistical analyses

The general characteristics of the participants were examined according to subject groups (the NCI-to-NCI non-conversion group vs. the NCI-to-MCI conversion group) using the chi-square tests and independent t-test as appropriate. Then, a repeated measures analysis of variance (repeated measures ANOVA) was conducted in order to examine the interaction between group and time with respect to the change in plasma HSPs level throughout the follow-up period. To minimize the potential confounding effects, the following covariates were entered into the adjusted model: age, gender, years of education, duration of follow-up, cerebrovascular risk factors (hypertension and diabetes), and APOE e4 allele status.

Linear mixed models were used to estimate the association between the plasma HSP level and cognitive change during the follow-up period. These models were applied separately based on plasma HSP 70 and HSP 27 levels. The linear mixed model included terms for time, plasma HSPs levels, age, gender, years of education, and the duration of follow-up. This model was subsequently expanded to include cerebrovascular risk factors (hypertension and diabetes) and APOE e4 allele status as covariates. A value of *p*<0.05 was considered statistically significant. SPSS software, version 18.0 (SPSS Inc., Chicago, IL, USA) was used for all analyses.

## Results

### 1. Baseline clinical characteristics

Clinical characteristics of the subjects according to the presence of conversion from NCI to MCI are described in Table 1. No significant differences were noted in the demographic and clinical characteristics between the conversion (N = 30) and non-conversion (N = 36) groups except years of education and follow-up MMSE scores (years of education: conversion group<non-conversion group, t = 2.18, *p* = 0.033; follow-up MMSE score: conversion group<non-conversion group, t = 4.72, *p*<0.001).

**Table 1:** Demographic and clinical characteristics of subjects. ^†^

		NCI to NCI	NCI to MCI	t or X	*p*
		non-conversion group (N = 36)	conversion group (N = 30)		
Age (year)	baseline	70.00±5.01	69.93±4.42	0.06	0.955
Gender female (N, %)		23 (63.89)	24 (80.00)	2.07	0.180
Education (year)		6.4±4.8	3.9±4.3	2.18	0.033
Follow up duration (year)		5.36±0.68	5.23±0.56	0.82	0.417
APOE e4 carrier (N, %)		7 (20.00)	5 (17.20)	0.78	0.778
HTN (N, %)		21 (58.33)	17 (56.67)	0.02	0.545
DM (N, %)		2 (5.56)	5 (16.67)	2.13	0.231
BMI (kg/m^2^)	Baseline	25.69±3.61	25.39±4.61	0.26	0.794
	Follow-up	25.87±3.81	25.83±4.61	0.04	0.972
K-MMSE	Baseline	26.89±2.27	26.17±2.37	1.26	0.211
	Follow-up	27.47±2.04	23.17±4.64	4.72	<0.001
K-SGDS	Baseline	5.14±4.07	6.38±4.03	-1.23	0.224
	Follow-up	5.97±5.12	8.43±6.74	-1.69	0.097

^†^ T-test or X^2^ test were used. All values are mean±SD (continuous variables) or frequencies (N, %) (categorical variables).

Abbreviation: NCI = no cognitive impairment, MCI = mild cognitive impairment, HTN = Hypertension, DM = Diabetes Mellitus, BMI = Body Mass Index, K-MMSE = Korean version of the Mini Mental Status Examination, K-SGDS = Korean version of Short Form Geriatric Depression Scale.

### 2. Interaction between group and time on change in plasma HSPs level

The conversion and non-conversion groups showed no significant differences in plasma HSP 70 and 27 levels at baseline. On the other hand, there was a significant difference in HSP 70 level between the two groups at follow-up (t = -3.43, *p* = 0.002) (Table 2).

**Table 2:** Plasma heat shock protein 70 and 27 levels at baseline and follow-up. ^†^

		NCI to NCI	NCI to MCI	t	*p*
		non-conversion group	conversion group		
HSP 70 (ng/ml)	Baseline	1.49±0.30	1.56±0.23	-0.97	0.335
	Follow-up	3.33±2.22	7.14±5.74	-3.43	0.002
HSP 27 (ng/ml)	Baseline	31.32±23.94	34.08±26.34	-0.45	0.657
	Follow-up	55.17±86.89	81.83±118.35	-1.05	0.296

† All values are mean±SD.

Abbreviation: NCI = no cognitive impairment, MCI = mild cognitive impairment, HSP = heat shock protein.

**Fig 1:**
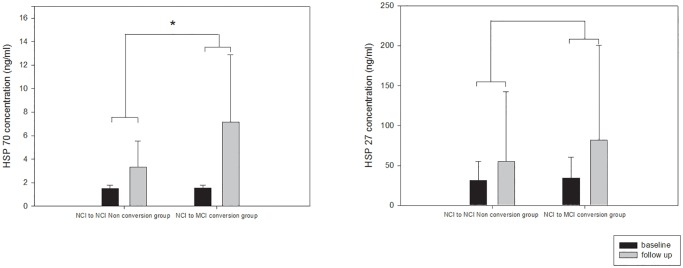
Interactive effects between time and group on change in plasma HSP levels. Repeated measure ANOVA was tested. Adjusted for age, gender, years of education, follow-up duration, APOE e4, hypertension, and diabetes (HSP70: F = 7.59 *p* = 0.008; HSP27: F = 0.67, *p* = 0.416). * *p* = 0.008.

Using repeated measure ANOVA, the interactive effects between the group and time on the change in plasma HSPs levels were tested after adjusting for confounding variables. We observed that plasma HSPs levels tended to increase with age, even though the change was not statistically significant (HSP 70: F = 0.07, *p* = 0.792; HSP 27: F = 0.25, *p* = 0.622). However, when considering the effect of the different groups, a significant interaction between group and time was observed in the plasma HSP 70 level (HSP 70: F = 7.59, *p* = 0.008; HSP 27: F = 0.67, *p* = 0.416) (Fig. 1).

### 3. Association between the change in HSPs levels and cognitive function during the follow-up period

Linear mixed model analyses showed that plasma HSP 70 level was negatively correlated with cognitive performance on the cognitive tests (K-BNT:—Coefficient [95% CI] -0.443 [-0.804, -0.081], *p* = 0.016; SVLT-delayed recall: -0.093 [-0.163, 0.022], *p* = 0.010; RCFT-copy: -0.174 [-0.707, 0.359], *p* = 0.522; COWAT: -0.171 [-0.329, -0.012], *p* = 0.035; Stroop: -0.278 [-0.447, -0.109], *p* = 0.001) after adjusting for age at baseline, gender, years of education, and follow-up duration. Even when the confounding effects of the APOE e4 allele, hypertension, and diabetes were considered together, the overall results were unchanged (K-BNT: -0.426 [-0.781, -0.071], *p* = 0.019; SVLT-delayed recall: -0.063 [-0.138, 0.012], *p* = 0.101; RCFT-copy: -0.041 [-0.569, 0.486], *p* = 0.877; COWAT: -0.176 [-0.328, -0.023], *p* = 0.024; Stroop: -0.304 [-0.458, -0.150], *p*<0.001). However, these associations were not statistically significant in relation to the plasma HSP 27 level. These findings are summarized in Table 3.

**Table 3:** Association between the change in heat shock protein level and cognitive function during follow-up period. ^†^

Dependent variables		Coefficient	95% C.I.	*p*
HSP 70				
Language function	K-BNT	-0.426	[-0.781, -0.071]	0.019
Memory function	SVLT-recall	-0.063	[-0.138, 0.012]	0.101
Visuospatial function	RCFT-copy	-0.041	[-0.569, 0.486]	0.877
Frontal/executive function	COWAT	-0.176	[-0.328, 0.023]	0.024
Frontal/executive function	Stroop test	-0.304	[-0.458, -0.150]	<0.001
HSP 27				
Language function	K-BNT	-0.014	[-0.028, 0.000]	0.051
Memory function	SVLT-recall	-0.001	[-0.006, 0.004]	0.679
Visuospatial function	RCFT-copy	0.003	[-0.014, 0.020]	0.733
Frontal/executive function	COWAT	-0.006	[-0.013, 0.001]	0.078
Frontal/executive function	Stroop test	-0.002	[-0.010, 0.007]	0.693

† Linear mixed model was tested.

Adjusted by age, gender, years of education, follow-up duration, APOE e4, hypertension, and diabetes.

Abbreviation: K-BNT = Korean version of Boston Naming Test, SVLT = Seoul Verbal Learning Test, RCFT = Rey-Complex Figure Test, COWAT = Controlled Oral Word Association Test.

## Discussion

In this study, we investigated the relationship between plasma HSP 70 and 27 levels and cognitive decline in the community-dwelling elderly. The results from our study showed that only the plasma HSP 70 level was associated with a conversion from NCI to MCI after adjustments for age, gender, years of education, follow-up duration, APOE e4 status, hypertension, and diabetes. Furthermore, an increased plasma HSP 70 level was associated with cognitive decline in language and executive functions.

An underlying mechanism of the linkage between increasing plasma HSP 70 level and the conversion from NCI to MCI has not yet been clearly identified, but the contribution of a cytoprotective mechanism seems plausible. Increasing oxidative stress markers have been reported in subjects with MCI [17–20]. Oxidative stress could be an early event of cognitive decline in the elderly [21,22]. The protein structure changes caused by oxidative damage generally result in impaired function and lead to the formation of protein aggregates in the cytosol [2]. HSP 70 is involved in the ubiquitin—proteasome pathway and is induced to defend against oxidative stress in its cytoprotective capacity [2,23]. For instance, HSP 70 was identified as a protector against Aβ accumulation and its overexpression rescued neurons from Aβ-mediated neurotoxicity [24,25].

Longitudinal analyses demonstrated increasing plasma HSP 70 levels were associated with a cognitive decline in language and executive functions, even after adjusting for confounding factors. A similar tendency was also observed in memory function before adjusting for the APOE e4 factor (SVLT-delayed recall: -0.093 [-0.163, 0.022], *p* = 0.010). However, the statistical significance of this association disappeared after adjustment for the presence of APOE e4 (SVLT-delayed recall: -0.063 [-0.138, 0.012], *p* = 0.101). The aggravation of cerebrovascular pathology in the elderly was known to be more closely related to language and executive functions than memory function [26–29]. Several studies have indicated that the expression of HSP 70 reduced ischemic injury, induced the inflammatory response, and protected both neurons and glial cells [10,30–34]. Taken together, our data present the possibility that an increase of plasma HSP 70 level may reflect functional declines in the cognitive domains known to be more closely associated with cerebrovascular injury.

Further, our results revealed that the plasma Hsp27 level did not show a significant relationship with the conversion to MCI. However, inverse correlation tendency between the plasma HSP 27 level and cognitive decline was observed, even though it was not statistically significant (K-BNT: -0.014 [-0.028, 0.000], *p* = 0.051; COWAT: -0.006 [-0.013, 0.001], *p* = 0.078). This may be due to the characteristics of HSP 27 function, which are modulated by its phosphorylation status. Several studies showed that HSP 27 directly bound to hyperphosphorylated tau without ubiquitination. Increased HSP 27 levels might be favored by the occurrence of hyperphosphorylated tau, which could protect the subsequent formation of the neurofibrillary tangle (NFT) [8,35–38]. NFT formation shows a tendency to increase gradually throughout the course of the illness unlike other neurodegenerative pathology, such as amyloid deposition which reaches a threshold early in the disease process [39]. Given that our subjects were in the relatively early phase of cognitive decline, the pathological state of our subjects might not have been enough to reach the conditions for HSP 27 overexpression.

There are several limitations to this study. First, the small sample size limits the predictive power of the study. First, the small sample size limits the predictive power of the study. GDEMCIS was a large scale community study but the current study only involved 66 subjects. To increase the certainty of diagnosis and exclude confounding factors in relation between HSPs level and early phage of cognitive declines in the elderly, we could include only a small number of subjects who had complete blood sample data and clinical follow-ups from the whole cohort. Therefore, these aspects might limit the representativeness of our sample as a community-based population. Second, there was only one follow-up measurements reported in this study. MCI has not been known as a confirmative condition. As people with MCI would improve or remain stable in cognition over time, it would be difficult to state any firm conclusion based on the findings in converters to MCI at only one point of follow up assessment. Third, the change in follow up HSPs levels might reflect a nonspecific disease state. Although we adjusted for the possible confounding factors, it should be noted that the observed interrelationship might not be a direct cause—effect relationship due to complex interactive factors, such as medications and age-related physical illnesses. Fourth, it is conceivable that plasma levels do not adequately reflect tissue levels or tissue responses. Fifth, analyses on association with different cognitive domains alone may not give direct evidences for the effect of HSPs on the degenerative process. The function of HSPs may be related nonspecific physiological effects on homeostasis and neuroprotection. So, it is necessary to carry out further investigations to prove out this relationship. Finally, it is possible that our subjects with MCI are a pathologically heterogeneous group. However, multiple pathologies might be more common than a single pure pathology in older adults with dementia [40].

Nonetheless, our study has several strengths. To our knowledge, this is the first study to report the temporal relationship between plasma HSP 70 levels and the conversion from NCI to MCI. We also uniquely demonstrated that language and executive function declines were associated more strongly with the change in plasma HSP 70 level in the elderly. This study raises the need for further investigations about HSPs involvement in the pathogenesis and progression of cognitive decline in the elderly.
